# Preparation and Compression Behavior of High Porosity, Microporous Open-Cell Al Foam Using Supergravity Infiltration Method

**DOI:** 10.3390/ma17020337

**Published:** 2024-01-10

**Authors:** Yuan Li, Zhe Wang, Zhancheng Guo

**Affiliations:** State Key Laboratory of Advanced Metallurgy, University of Science and Technology Beijing, Beijing 100083, China; b20200585@xs.ustb.edu.cn (Y.L.); zhewang@ustb.edu.cn (Z.W.)

**Keywords:** supergravity infiltration, high porosity Al foam, compression behavior

## Abstract

By employing a method that combines a NaCl compacting template and supergravity infiltration, open-cell aluminum (Al) foam with varying porosities was prepared. The Al foam fabricated has a pore size of 400 µm and porosity ranging from 0.72 to 0.88. The experimental results indicate that, with an increase in compaction pressure during the NaCl compacting process, the porosity of the foam Al increases and the struts become finer. As the gravity coefficient increases, the density and integrity of the foam Al also increase. Due to the effectiveness of supergravity in overcoming the infiltration resistance between the NaCl preform and molten Al, the supergravity infiltration method holds promise as a practical new technique for fabricating high-porosity open-cell Al foam.

## 1. Introduction

Porous metallic foams, as versatile engineering materials with dual outstanding properties in both functionality and structure, have rapidly become a prominent new material in the current field of materials science [1,2]. In comparison to solid structural materials, porous metallic foams are not only lightweight with excellent mechanical properties but also exhibit remarkable energy absorption properties [3,4]. Aluminum (Al) foam is the most common and widely used metal foam [5,6]. It consists of pure Al or Al alloy, featuring a multitude of three-dimensional pore structures. In contrast to common dense metals, Al foam demonstrates superior impact resistance and higher specific strength. Furthermore, it possesses excellent acoustic, energy absorption and heat exchange properties. Therefore, it has a very high potential for applications in the automotive, marine, transportation, military and aerospace fields [7,8,9].

At present, the most common methods for preparing Al foam are melt foaming and infiltration casting [10], of which the melt foaming method has the advantages of low cost and simple operation. However, it is often contradictory in this method to simultaneously increase porosity and decrease pore size. That is, as porosity increases, pore size also tends to increase. Therefore, it is challenging to produce Al foam that possesses both high porosity and a small pore size at the same time [11,12]. The basic principle of the infiltration casting method is to allow the liquid metal to infiltrate into the interstices of the porous preform, solidify and cool, and then remove the filler particles using appropriate methods to obtain the final metal foam. Because the liquid metal and the preform is often not wetted, the liquid metal cannot spontaneously infiltrate the voids of the preform under the normal gravity field. To overcome the resistance to infiltration caused by the surface tension of the liquid metal, it is common to adopt methods such as pressurization, using vacuum or a combination of both to promote the flow of liquid metal in the interstices of the particles and increase the length of infiltration. Currently, the majority of open-cell Al foam has a porosity below 0.8. Bhasker Soni et al. [13] prepared a 6061 Al alloy foam with porosity ranging from 0.5 to 0.75 via the pressure infiltration method, and investigated both the porosity and mechanical properties of the samples with different porosities. Xia et al. [14] prepared open-cell Al foam with a porosity of 0.65–0.77 and a pore size of 0.15 mm–0.6 mm using vacuum infiltration with NaCl as a preform. However, higher porosity microporous Al foam is less reported due to the increased difficulty of preparation as porosity rises and pore size decreases. This requires the application of greater additional pressure to drive the infiltration of the liquid metal, leading to increased equipment requirements. Al foam with a smaller pore size and higher porosity possesses a larger specific surface area and lower relative density. Therefore, there is a greater demand for applications in fields such as catalysis, heat exchange, sound absorption, decoration and others. Based on this, the present study intends to utilize the supergravity infiltration method to prepare Al foam with a fine pore size and high porosity.

Supergravity is a force field created by centrifugal rotation that can exert significant additional pressure to facilitate the penetration of metallic fluids as the rotational speed increases [15,16]. This force can be harnessed to overcome the resistance to infiltration caused by the surface tension of the previously mentioned liquid Al. The intense shear stress induced by supergravity makes the surface tension of the liquid negligible, effectively encouraging the liquid metal to infiltrate into pores of various sizes within the supergravity field. This capability enables the preparation of three-dimensional foam metals with various pore sizes, and the infiltration rate and length can be controlled effectively by adjusting the coefficient of supergravity. Under the influence of the supergravity field, the infiltration process of the metallic liquid becomes more robust, leading to a significant reduction in the required temperature and time for infiltration. Therefore, supergravity not only enhances the production efficiency of foam metal but also reduces the cost of the preparation process. Currently, the preparation of foam metals and metal matrix composites through supergravity infiltration has become a practical process.

In this study, NaCl particles were compacted to create high-volume fraction preforms. Subsequently, Al foam with high porosity and a small pore size was prepared using the supergravity infiltration method. And the study further investigates the influence of the physical properties and gravity coefficient of the preform on the microstructure and mechanical properties of the prepared foam aluminum. Additionally, the impact of supergravity on the infiltration velocity is clarified through calculations.

## 2. Experimental

### 2.1. Materials

In this study, 99.99% pure Al was utilized as the base metal, and commercially available NaCl particles with a particle size of 0.3–0.5 mm were used to prepare the preform.

### 2.2. Centrifugal Infiltration

**Figure 1:**
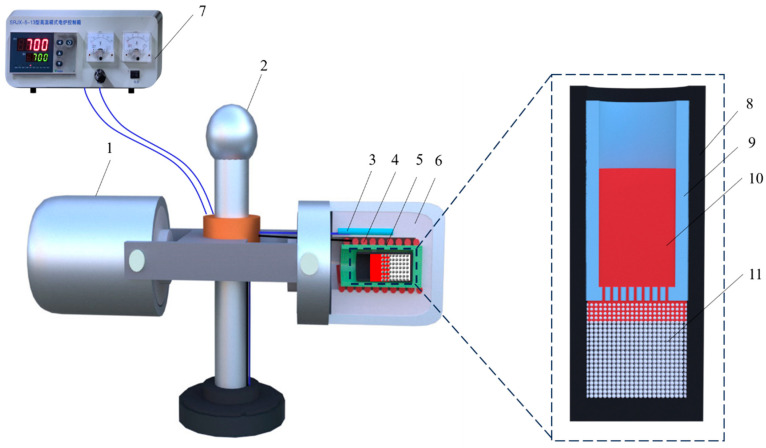
Schematic showing centrifugal force apparatus used in this work: (1) counterweight, (2) centrifugal axis, (3) thermocouple, (4) resistance coil, (5) cylindrical corundum chamber, (6) alumina insulation cavity, (7) temperature controller, (8) internal crucible, (9) outer crucible, (10) molten Al and (11) NaCl particles.

On Earth, supergravity is generated through centrifugal rotation. As shown in Figure 1, the centrifuge used in this study has been modified from a medical refrigerated centrifuge (DL-8M, Luxiangyi Co., Ltd., Shanghai, China). To maintain balance, heating furnaces and counterweight furnaces are installed on both sides of the rotating axis. The heating furnace consists of a stainless steel outer shell, alumina insulation cavity, resistance wire, alumina heating tube and a K-type thermocouple. The maximum heating temperature is 1350 °C, with a heating range of a diameter of 40 mm and a length of 150 mm. The temperature observation accuracy is within ±3 °C.

The gravity coefficient (G) represents the magnitude of centrifugal force and is defined as the ratio of centrifugal force to the standard gravitational force.

(1) $$G=\frac{N^{2}\pi^{2}R}{900g}$$

where *N* represents the rotation speed of the centrifuge; *R* represents the length of the centrifugal arm (in this study, *R* = 0.25 m); and *g* represents the standard gravitational acceleration, 9.8 m/s².

**Figure 2:**
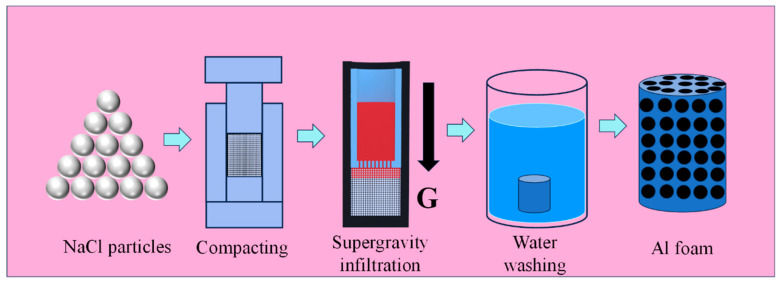
Experimental flowchart for the preparation of Al foam using supergravity infiltration method.

**Figure 3:**
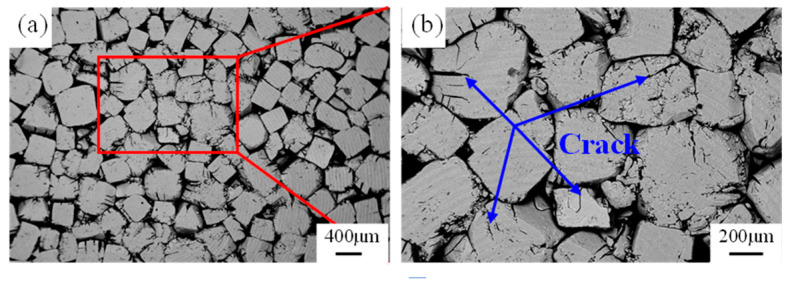
The microstructure of the compressed NaCl preform, (**a**) microstructure, (**b**) magnification of the boxed area in (**a**).

The experimental flowchart for preparing foam aluminum using the supergravity infiltration method is shown in Figure 2. The preparation of high-porosity open-cell Al foam in this study mainly involves three steps: (1) the preparation of the preform, (2) supergravity infiltration and (3) water washing. First, NaCl particles were dried in an oven at 120 °C for 4 h to remove free water from the NaCl particles. Using a stainless steel mold, approximately 35 g of the mixture was compressed into a cylindrical preform with a diameter of 30 mm and a height of about 30 mm (the height varied under different pressures). The compaction pressure is as shown in Table 1. The geometric morphology of the preform is shown in Figure 3, and Figure 3b is an enlargement of the boxed area in Figure 3a. It can be observed that the NaCl particles exhibit irregular cubic particle structures, with compact spacing between particles. As shown in Figure 3b, during the compression molding process, many NaCl particles were compressed and fractured, resulting in numerous cracks as indicated by arrows. These cracks are likely to be filled during the subsequent infiltration process.

As shown in Figure 1, the crucible used in the experiment is a self-designed combination crucible with inner and outer components. The bottom of the inner crucible has several small holes with a diameter of 0.5 mm. This combination crucible was employed to prevent the NaCl preform from floating in the molten Al under the influence of supergravity (the density of NaCl (2165 kg/cm^3^) is lower than the density of molten Al (2700 kg/cm³)). The prepared NaCl preform was placed in the outer crucible, while Al granules were placed in the inner crucible, forming a complete experimental crucible. The experimental crucible was then placed in the heating furnace of the centrifuge and heated to 700 °C at a rate of 7 °C/min, with a 20 min dwell time to ensure the complete melting of the Al. Finally, the centrifuge was activated to allow the molten Al to infiltrate the pores of the NaCl preform under different gravity conditions, as shown in Table 1. After centrifugation for 5 min, the centrifuge and heating furnace were turned off. After cooling to room temperature, the sample was removed and immersed in water to remove the NaCl particles, resulting in the formation of open-cell Al foam. 

### 2.3. Characterization

The microstructures of NaCl particles and Al foam prepared under different experimental conditions were observed using a scanning electron microscope (SEM, MLA 250, FEI Quanta, Houston, TX, USA). The relative density ( $\rho_{d}$ ) of the sample was calculated by Equation (2):

(2) $$\rho_{d}=\left(\frac{\rho_{c}}{\rho_{s}}\right)\times 100\%$$

where $\rho_{s}$ denotes the density of metallic Al and $\rho_{c}$ denotes the density of Al foam, measured using the volumetric density method. The porosity ( ϕ ) of Al foam is calculated using Equation (3):

(3) $$\phi =1-\rho_{d}$$

Compression performance tests were conducted on the Al foam samples using a universal testing machine, with sample dimensions of a diameter of 20 mm and a height of 25 mm. Each experimental group was repeated three times to reduce experimental errors.

## 3. Results 

### 3.1. Effect of Compaction Pressure on NaCl Preform

**Figure 4:**
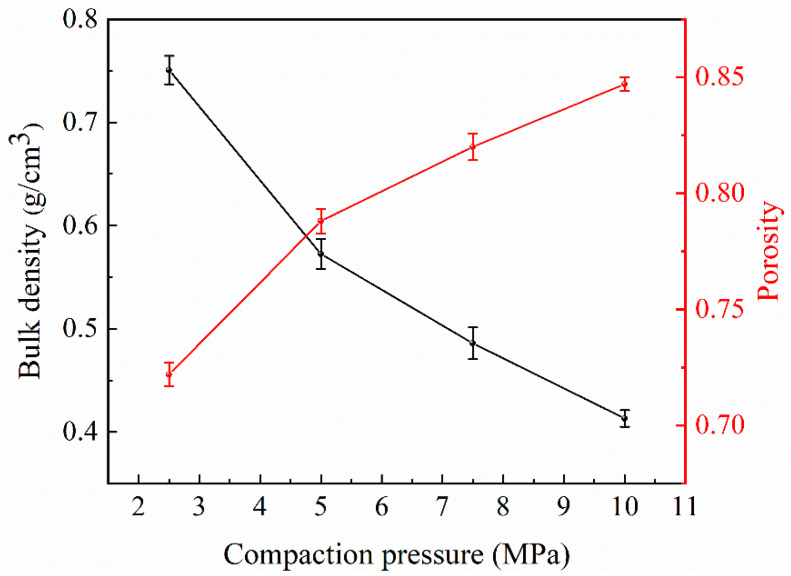
The bulk density and porosity of Al foam prepared from NaCl preforms compacted under different pressures (G = 1000).

**Figure 5:**
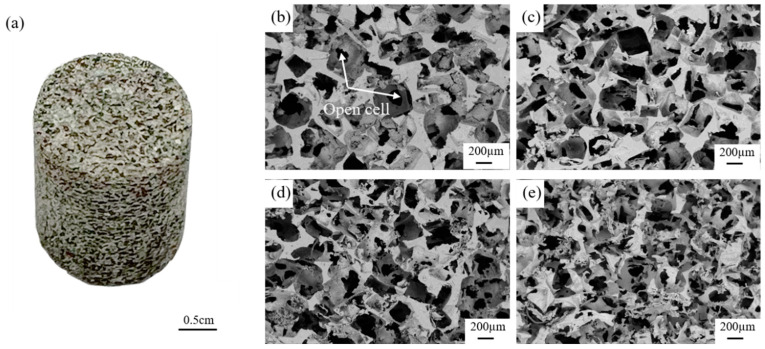
The macroscopic morphology of Al foam and the microscopic morphology of Al foam prepared from NaCl preforms compacted under different pressures, (**a**) macro-photography of the sample, (**b**) 2.5 MPa, (**c**) 5 MPa, (**d**) 7.5 MPa and (**e**) 10 MPa (G = 1000).

As shown in Figure 4, the bulk density of Al foam decreases with the increase in compaction pressure of NaCl preforms, while the porosity increases in the opposite direction. The highest porosity of Al foam prepared by compacting NaCl preforms at a pressure of 10 MPa is 0.847. As shown in Figure 5, the macroscopic morphology of Al foam prepared using the supergravity infiltration method reveals the uniform distribution of numerous irregularly shaped pores on the surface of the Al foam. These pores correspond to the spaces initially occupied by NaCl. Figure 5b–e, respectively, show the microscopic morphology of Al foam prepared by compacting NaCl preforms with pressures of 2.5 MPa, 5 MPa, 7.5 MPa and 10 MPa. It can be observed that the pore shapes are consistent with the shapes of NaCl particles, indicating that the prepared Al foam perfectly replicates the pores of the NaCl preform. There are some micropores on the cell walls of Al foam prepared under different conditions, connecting the pores of the Al foam and providing continuity. When the compaction pressure is low at 2.5 MPa, the columns of Al foam are thicker, and there are fewer interconnected openings between the cell walls. The surface of the cell walls is relatively smooth. As the compaction pressure increases from 2.5 MPa to 10 MPa, the columns of Al foam gradually become finer, and the interconnected openings between the cell walls increase. This is mainly because, during the compaction of NaCl, the contact area between adjacent NaCl particles gradually increases, and these areas become the openings on the cell walls of the prepared Al foam. This is conducive to improving the permeability of Al foam.

**Figure 6:**
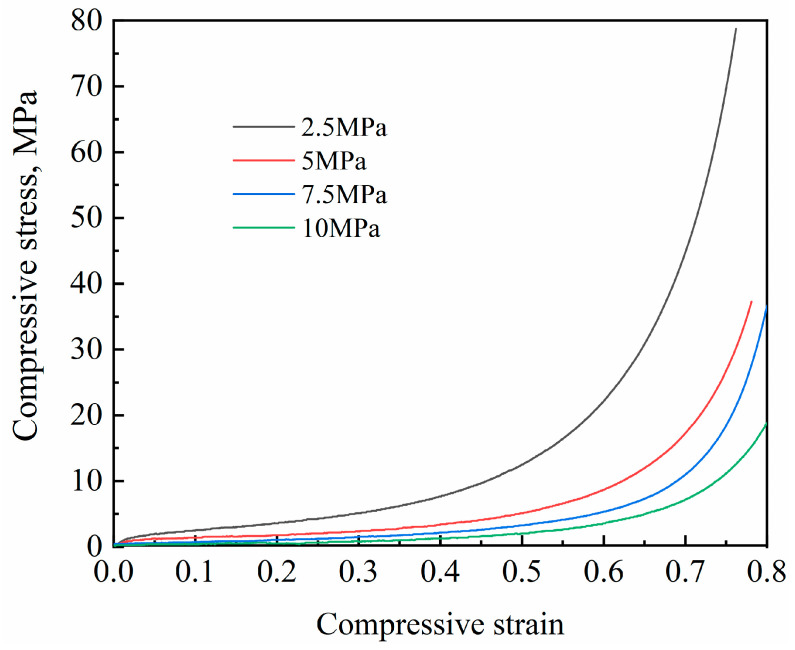
The stress–strain curves of Al foam prepared by compacting NaCl preforms under different pressures.

The stress–strain curves of Al foam prepared by compacting NaCl preforms under different pressures are shown in Figure 6. Similar to conventional foam metals [17], the stress–strain curve of Al foam prepared using the supergravity infiltration method can be divided into three typical stages: the initial elastic stage, the plastic plateau stage and the densification stage. At the beginning of compression, stress increases linearly with strain, forming the elastic stage. In the plateau stage, stress increases slowly with increasing strain, exhibiting significant and stable hardening behavior. During this stage, the cell walls of Al foam deform and collapse under the influence of stress. With further strain, the pores of Al foam are gradually compressed, and stress sharply increases with strain, leading to the densification stage. It can be observed that, when the compaction pressure on the NaCl preform is low, the elastic stage is more pronounced. As the compaction pressure on NaCl particles increases to 7.5 MPa, the prepared Al foam has a lower relative density, higher porosity and further decreased mechanical properties. 

Table 2 shows the bulk density (ρ_c_), porosity (*ϕ*), yield strength (σ_s_), plateau stress (σ_pl_) and average densification strain (ε_D_) of Al foam prepared under different conditions. The yield strength is considered as the mean value of stress at ε = 0.05. The plateau stress represents the average stress measured between the elastic and densification stages. The densification strain is calculated using the two-tangent method. It can be observed that, with an increase in the compaction pressure of the preforms, the mechanical properties of Al foam, including yield strength and plateau stress, decrease, while the densification strain increases. This is mainly attributed to the decrease in the relative density of Al foam.

### 3.2. Effect of Gravity Coefficient

**Figure 7:**
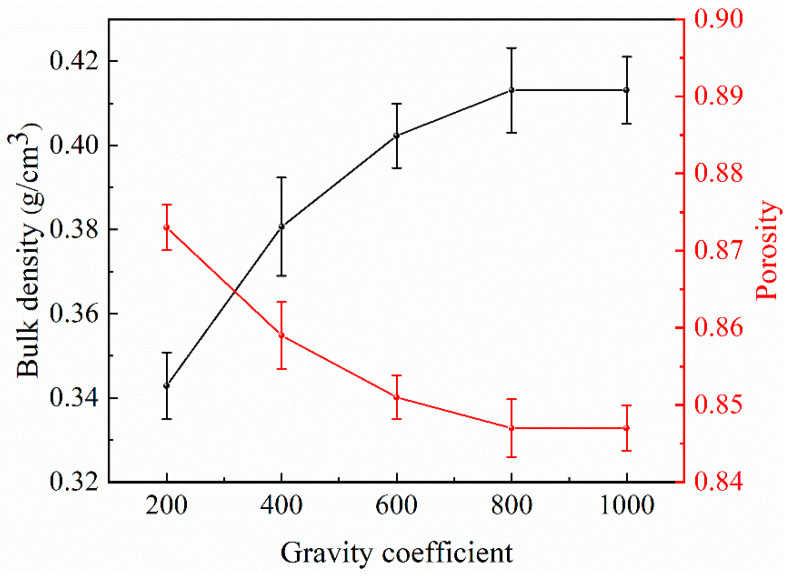
The bulk density and porosity of Al foam prepared under different gravity coefficients (compaction pressure = 10 MPa).

The variation in bulk density and porosity of Al foam prepared under different gravity coefficients is shown in Figure 7, with a compaction pressure of 10 MPa applied to the NaCl preforms. It can be observed that the bulk density of the foam increases with the increment of the gravity coefficient, while the porosity exhibits the opposite trend. When the gravity coefficient is relatively low, at G = 200, the bulk density of the Al foam is 0.342 g/cm³, with a porosity reaching its maximum value in this study at 0.873. It is noteworthy that the chosen gravity coefficient of 200 for this experiment is due to the limitation where Al cannot infiltrate the pores of the NaCl preforms at lower gravity coefficients. As the gravity coefficient increases to 400, the bulk density of Al foam rapidly rises to 0.381 g/cm^3^, with a porosity of 0.859. When the gravity coefficient further increases to 600, the bulk density increases to 0.402 g/cm^3^, and the porosity decreases to 0.851, with a noticeable reduction in the rate of increase. When G = 800, the bulk density of Al foam reaches its maximum at 0.413 g/cm^3^, with a porosity of 0.847. As the gravity coefficient continues to increase to 1000, the change in bulk density becomes negligible, indicating that the Al has completely filled the pores of the NaCl preforms.

**Figure 8:**
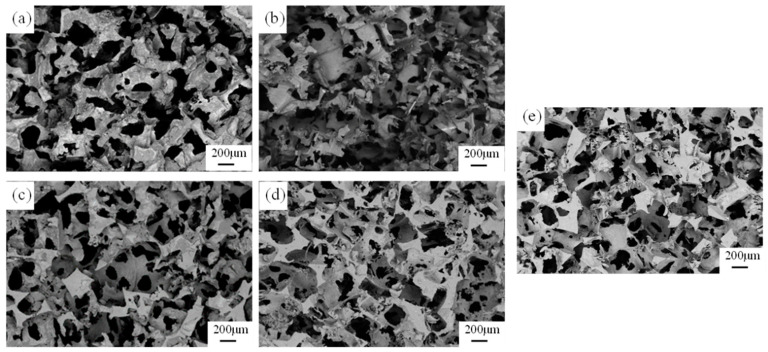
The microscopic morphology of Al foam prepared under different gravity coefficients: (**a**) G = 200 G, (**b**) G = 400, (**c**) G = 600, (**d**) G = 800 and (**e**) G = 1000 (compaction pressure = 10 MPa).

The SEM images of Al foam prepared under different gravity coefficients are shown in Figure 8, where (a), (b), (c), (d) and (e) correspond to gravity coefficients of 200, 400, 600, 800 and 1000, respectively. When the gravity coefficient is 200, the connecting struts between the cell walls of the Al foam are very fine, and there are many missing cell walls. As the gravity coefficient increases, the struts gradually become more robust, and the number of cell walls increases. It is worth noting that, at higher gravity coefficients, some protruding thin sheets or struts appear on the cell walls of the prepared Al foam. Studies by Zhang and Cheng [18] suggest that, in low-frequency sound waves, the sound absorption coefficient of foam metals is positively correlated with the surface impedance. The formation of fine Al dendrites on the surface of Al foam prepared at higher gravity coefficients is conducive to its acoustic performance.

**Figure 9:**
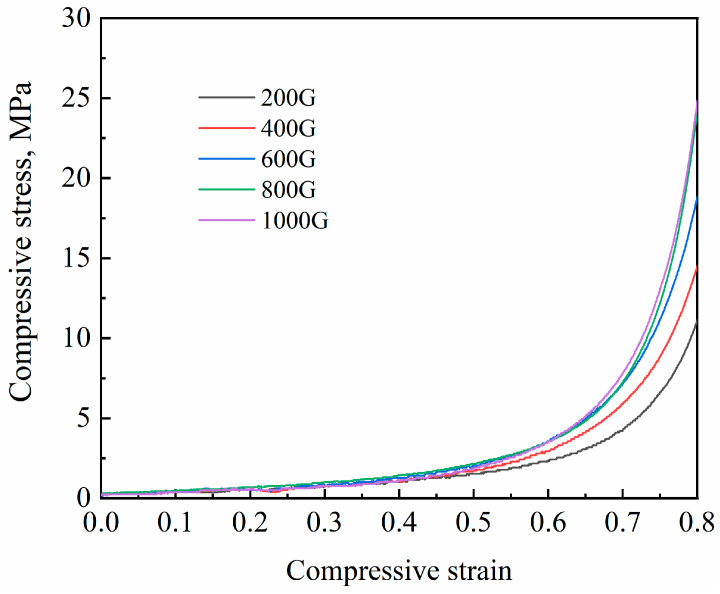
The stress–strain curves of Al foam prepared under different gravity coefficients.

The strain–stress curves of Al foam prepared under different gravity coefficients are depicted in Figure 9, showing similar trends. Particularly in the elastic and plateau stages, the stress–strain curves of Al foam prepared under different gravity coefficients almost overlap. It is only in the densification stage that the stress of Al foam at the same strain increases with the increment of the gravity coefficient. The mechanical properties of Al foam prepared under different gravities are as shown in Table 3.

## 4. Discussion 

### 4.1. Compressive Behavior Sensitivity

The relative density of porous aluminum foam is a primary factor influencing its mechanical performance. Therefore, the mechanical performance indicators of aluminum foam prepared using NaCl templates with different gravity coefficients and compaction pressure degrees are determined by the relative density. The Gibson–Ashby model is used to describe the relationship between the bulk density and plateau stress of foam materials, as shown in Equation (4) [19,20]:

(4) $$\frac{\sigma_{pl}}{\sigma_{ys}}=C{\left(\frac{\rho_{c}}{\rho_{s}}\right)}^{n}$$

where *σ_ys_* represents the compressive yield strength of the base material aluminum; *C* is a proportionality constant influenced by pore geometry; and n is the density exponent associated with deformation mechanisms, which is typically around 1.5. Taking the logarithm of both sides of Equation (4) results in Equation (5).

(5) $$\log\sigma_{pl}=n\log\rho_{s}+C^{\prime }$$

**Figure 10:**
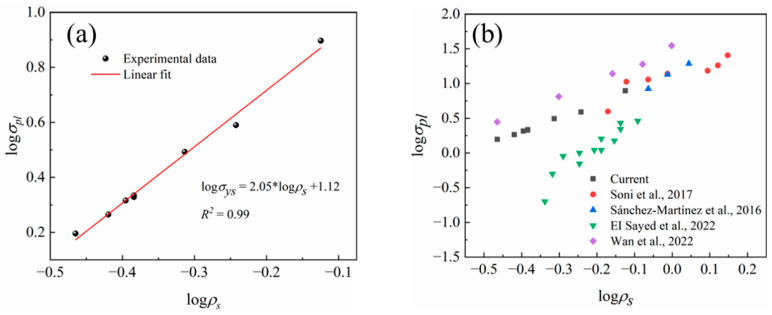
The relationship between plateau stress and relative density of Al foam in both the current study and other studies, (**a**) Linear fitting for the current study, (**b**) other studies [13,21,22,23].

By plotting the Al foam samples obtained under different conditions according to Equation (5), the relationship between plateau stress and sample density was established, as shown in Figure 10a. The experimentally fitted correlation coefficient (R^2^) is 0.99, indicating that the plateau stress of the samples obtained under all conditions conforms to this model. This is because, as depicted in Figure 8, with a decrease in Al foam density, the foam’s cell walls decrease, and more pore structures serve as pillars, aligning closely with the Gibson–Ashby model. However, the slope of the fitted line is higher than that of the Gibson–Ashby model, suggesting that the compression behavior of Al foam prepared using the centrifugal infiltration method is more sensitive. This is primarily due to a deviation in the definition of *σ_pl_* between this study and the Gibson–Ashby model. In the Gibson–Ashby model, the plastic plateau region of the stress–strain curve for foam samples is a distinct horizontal segment. The yield strength of open-cell foam is defined as the complete plastic collapse of cell structures. In this study, however, the stress in the plastic plateau region gradually increases with increasing strain, and the stress–strain curve of the sample does not exhibit a clear elastic stage limit and peak stress. Therefore, the plateau stress (*σ_pl_*) is used to represent the plastic collapse. On the other hand, a small number of cell walls and struts form a mixed strut structure, resulting in a higher compressive strength at the same density compared to the Gibson–Ashby model [14].

Figure 10b illustrates the relationship between the relative density and plateau stress of Al foam in both the current study and other studies [13,21,22,23]. Table 4 provides the specific process parameters and performance indicators from these references. The compressive sensitivity of Al foam reported in various sources in the literature is consistently higher than predicted by the Gibson–Ashby model. Al foams with similar relative densities exhibit similar plateau stresses. It is evident that the quality of the Al foam prepared using the supergravity infiltration method in this study is comparable to that of Al foam prepared using other methods.

### 4.2. Supergravity Infiltration

**Figure 11:**
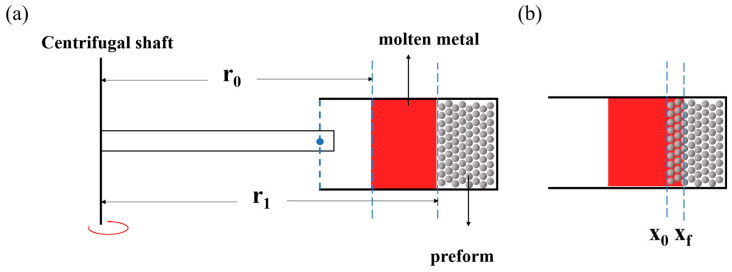
Diagram illustrating the infiltration process, (**a**) before infiltration, (**b**) during the infiltration process.

Due to the non-wetting nature between Al and NaCl, Al cannot spontaneously infiltrate the pores of the NaCl preforms. This requires a centrifugal pressure greater than the capillary resistance caused by the non-wetting behavior. The centrifugal pressure applied to the NaCl preforms during the experimental process can be calculated using Equation (6):

(6) $$P_{c}=\frac{\rho_{m}\omega^{2}(r_{1}^{2}-r_{0}^{2})}{2}=\frac{\rho_{m}N^{2}\pi^{2}(r_{1}^{2}-r_{0}^{2})}{1800}$$

where *ρ_m_* represents the density of molten Al, 2380 kg/m^3^ at 700 °C, and *ω* denotes the magnitude of angular velocity during the centrifugal process. *r*_0_ and *r*_1_ represent the distances from the centrifugal axis to the inner and outer surfaces of the NaCl preform, as shown in Figure 11a, respectively, being 0.22 m and 0.26 m. The minimum additional pressure required for metal liquid infiltration is referred to as the infiltration threshold pressure (*P_th_*), which can be expressed by Equation (7) [24,25]:

(7) $$P_{th}=-S_{i}\sigma_{\mathrm{lg}}\cos\theta$$

where *S_i_* represents the interfacial area of the base metal per unit volume; *σ_lg_* represents the surface tension of the infiltrating metal, 0.873 N/m [26]; and *θ* is the contact angle between the NaCl preform and molten Al, 140° [27]. 

For particle-reinforced metal matrix composites, the reinforcing particles can be considered as equivalent spheres with the same volume, and their average diameter is denoted as *D_p_,* 400 μm. Thus, *S_i_* can be expressed by Equation (8):

(8) $$S_{i}=\frac{6\lambda V_{p}}{D_{p}(1-V_{p})}$$

where *V_p_* represents the volume fraction of reinforcing particles in the preform and *λ* is the shape factor, 2.95 [28], which is related to the shape and roughness of the particles. Combining Equations (7) and (8), the relationship between the infiltration threshold pressure and the particle preform can be obtained and expressed by Equation (9):

(9) $$P_{th}=-6\lambda \sigma_{\mathrm{lg}}\cos\theta \frac{V_{p}}{D_{p}(1-V_{p})}$$

At a low gravity coefficient, the molten Al can only infiltrate into the larger pores of the NaCl preform. As the gravity coefficient increases, molten Al infiltrates into the narrower spaces within the preform, resulting in an increase in the relative density and structural integrity of Al foam. 

The infiltration threshold pressure determines whether metal liquid can initiate infiltration under a certain additional pressure, but it does not address issues such as infiltration time and length. These problems can be calculated using Darcy’s law. Assuming the fluid flows unidirectionally through a porous medium, Darcy’s equation [7,8] can be expressed by Equation (10) [29,30]:

(10) $$v_{0}(t)=-\frac{K}{\mu }\frac{\partial P(x,t)}{\partial x}$$

where *v*_0_(*t*) is the average apparent velocity of the fluid; *x* is the direction of fluid flow; *∂P*(*x,t*)/*∂x* represents the pressure gradient in the direction of infiltration; *K* is the permeability of the porous medium; and *μ* is the viscosity of the fluid, 0.948 × 10^−3^ Pa∙s [31].

Since the preform is incompressible, with a fixed cross-sectional area, the *v*_0_(*t*) is independent of the *x*. The continuity equation is expressed as Equation (11):

(11) $$\frac{\partial v_{0}\left(t\right)}{\partial x}=0$$

At a fixed time t, Equation (10) can be regarded as a first-order linear differential equation with respect to x. When *x* = *x*₀ = 0, *P*(*r*, *t*) = *P*₁, representing additional pressure. When *x* = *x_f_*, *P*(*r*, *t*) = *P_th_*, indicating the infiltration resistance. As shown in Figure 11b, *x*_0_ and *x_f_* represent the initial position and the front position of infiltration, respectively. Integrating Equation (10) yields Equation (12):

(12) $$\int_{x_{0}}^{x_{f}}v_{0}(t)dx=\int_{P_{1}}^{P_{th}}-\frac{K}{\mu }dP(x,t)$$

Computing Equation (13) from Equation (12):

(13) $$v_{0}(t)x_{f}=\frac{K}{\mu }\Delta P$$

The average apparent velocity of the fluid can also be expressed by the following Equation (14):

(14) $$v_{0}(t)=(1-V_{p})\frac{dx_{f}}{dt}$$

Substituting Equation (14) into Equation (13) and simplifying, Equation (15) is obtained:

(15) $$(1-V_{p})x_{f}dx_{f}=\frac{K}{\mu }\Delta Pdt$$

Integrating Equation (15) yields Equation (16):

(16) $${x_{f}}^{2}=\frac{2Kt}{\mu (1-V_{p})}\Delta P$$

where Δ*P* represents the difference between centrifugal pressure and infiltration resistance, and can be calculated using the following Equation (17):

(17) $$\Delta P=P_{c}-P_{th}$$

The permeability (*K*) of granular particle-packed preforms can be calculated using the Kozeny–Carman formula, which is represented by Equation (18) [32]:

(18) $$K=\frac{\phi^{3}}{180{\left(1-\phi \right)}^{2}}{D_{p}}^{2}$$

**Figure 12:**
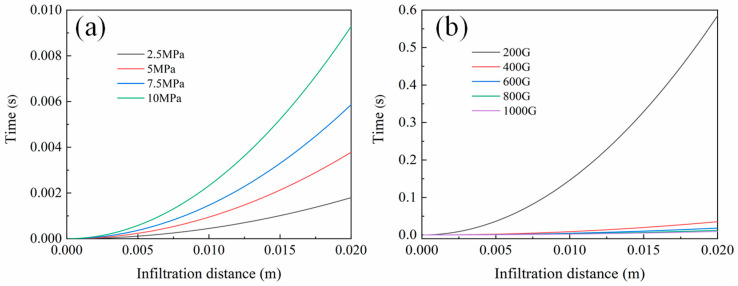
The relationship between the calculated infiltration distance and time according to Equation (16), (**a**) different compaction pressures, (**b**) different gravity coefficients.

As shown in Figure 12a, the relationship between the infiltration speed and time of foam Al prepared from NaCl preforms with different compaction pressures is depicted. It can be observed that, as the NaCl preform is compacted, its pores become finer, resulting in a slower infiltration speed of the molten aluminum. Meanwhile, the infiltration speed of molten aluminum accelerates with an increase in the gravity coefficient, as shown in Figure 12b. When the gravity coefficient is 200 G, it takes 0.59 s to infiltrate a 2 cm preform. As the gravity coefficient increases to 1000 G, the time shortens to 0.009 s, a 65-fold reduction. This indicates that an increase in gravity coefficient significantly hastens the infiltration speed of molten aluminum in the NaCl preform.

As the porosity of the preform decreases and the particle size decreases, the infiltration resistance gradually increases, requiring substantial additional pressure to overcome this obstacle. However, supergravity can precisely provide this additional pressure, forcing the metal liquid to infiltrate into the smaller pores of the preform, ultimately resulting in Al foam with a fine pore size and high porosity. Under high gravity coefficients, aluminum liquid can infiltrate into the cracks of NaCl particles, leading to a complex structure in the prepared Al foam, as illustrated in Figure 8.

In a supergravity field, the gravity coefficient can be adjusted by changing the rotation speed, thereby obtaining Al foam with different porosities to meet various usage conditions. Generally, with the increase in the gravity coefficient, Al foam becomes more complete, with an increase in relative density and improvement in the mechanical properties of the final sample. Moreover, the supergravity infiltration method can be used to produce nearly final-shaped porous Al foam, avoiding secondary processing and reducing production costs.

Current research suggests that the supergravity infiltration method is a highly promising industrial method for producing Al foam. This study investigates the physical properties of NaCl preforms and the impact of gravity coefficients on the microstructure and mechanical properties of Al foam. This can provide reference process parameters for the industrial production of Al foam using the supergravity infiltration method. However, the production of large-sized Al foam still requires the development of larger equipment, necessitating further research and exploration.

## 5. Conclusions

The main conclusions of this study are as follows:

(1) High-porosity aluminum foam with a porosity range of 0.72–0.88 was prepared using supergravity as an additional pressure and NaCl as a template. This method is characterized by its simplicity and has the potential for industrial-scale production.

(2) With an increase in the gravity coefficient, the struts of the foam Al become more robust and the cell walls gradually increase, leading to an increase in relative density and a decrease in porosity. An elevated gravity coefficient significantly enhances the infiltration speed of the molten metal, thereby improving production efficiency rapidly.

(3) The mechanical performance indicators of foam Al prepared using NaCl templates with different compaction pressures and under different gravity conditions are predominantly governed by relative density. These properties can be fitted and predicted using the Gibson–Ashby model.

## Figures and Tables

**Table 1:** Experimental conditions for the preparation of Al foam.

Group	Compaction Pressure, MPa	Gravity Coefficient
1	2.5	1000
2	5	1000
3	7.5	1000
4	10	1000
5	10	200
6	10	400
7	10	600
8	10	800

**Table 2:** The mechanical properties of Al foam prepared from NaCl preforms compacted under different pressures.

Compaction Pressure, MPa	Bulk Density (ρ_c_), g/cm^3^	Porosity (*ϕ*)	Yield Strength (σ_s_), MPa	Plateau Stress (σ_pl_), MPa	Densification Strain (ε_D_)
2.5	0.751 ± 0.014	0.722 ± 0.005	1.68 ± 0.008	7.88 ± 0.07	0.64 ± 0.003
5	0.572 ± 0.014	0.788 ± 0.005	1.02 ± 0.007	3.89 ± 0.06	0.67 ± 0.003
7.5	0.486 ± 0.015	0.820 ± 0.006	0.57 ± 0.006	3.11 ± 0.09	0.73 ± 0.004
10	0.413 ± 0.008	0.847 ± 0.003	0.38 ± 0.005	2.08 ± 0.02	0.74 ± 0.002

**Table 3:** The mechanical properties of Al foam prepared under different gravities.

Graity Coefficient	Bulk Density (ρ_c_), g/cm^3^	Porosity (*ϕ*)	Yield Strength (σ_s_), MPa	Plateau Stress (σ_pl_), MPa	Densification Strain (ε_D_)
200	0.343 ± 0.008	0.873 ± 0.003	0.26 ± 0.006	1.57 ± 0.03	0.76 ± 0.002
400	0.381 ± 0.011	0.859 ± 0.004	0.31 ± 0.002	1.84 ± 0.02	0.75 ± 0.003
600	0.402 ± 0.008	0.851 ± 0.003	0.36 ± 0.007	2.07 ± 0.04	0.74 ± 0.001
800	0.413 ± 0.010	0.847 ± 0.004	0.37 ± 0.008	2.16 ± 0.02	0.74 ± 0.003
1000	0.413 ± 0.008	0.847 ± 0.003	0.38 ± 0.005	2.08 ± 0.02	0.74 ± 0.002

**Table 4:** Process parameters and performance indicators of Al foam prepared in different studies in the literature.

References	Materials	Particle	Pore Size, mm	Relative Density	n in Equation (5)	Preparation Method
[13]	6061-T6 Al alloy	NaCl	0.75–2.5	0.25–0.52	1.91	Pressure infiltration
[21]	Zn-22Al-2Cu	NaCl	0.42–0.85	0.32–0.41	3.34	Centrifugal infiltration
[22]	A356 Al alloy	NaCl	2 and 4	0.19–0.3	3.92	Pressure infiltration
[23]	ZL102 Al alloy	Ca_2_Cl	3	0.13–0.39	2.3	Pressure infiltration
Current	Pure Al	NaCl	0.3–0.5	0.12–0.28	2.05	Supergravity infiltration

## Data Availability

Data available in a publicly accessible repository.
